# We could be better if we ate better

**DOI:** 10.20945/2359-3997000000262

**Published:** 2020-06-05

**Authors:** Licio A. Velloso

**Affiliations:** 1 Centro de Pesquisa em Obesidade e Comorbidades Universidade Estadual de Campinas SP Brasil Centro de Pesquisa em Obesidade e Comorbidades, Universidade Estadual de Campinas, SP, Brasil

When I was kindly asked by our editor-in-chief, Marcello Bronstein, to write an editorial about the article “Nutritional genomics, inflammation and obesity” ( 1 ), the first title that came to my mind was “We are what we eat”. So, I logged in PubMed and set the title in to browse for articles with similar wording and, to my surprise, there were 98 hits. So, for the sake of originality, I picked another title, as you see in the top of this text. I must say that I still prefer the original one I had in mind. However, the new title has the advantage of bringing hope. And hope is what we mostly need nowadays.

Obesity is affecting a rapidly growing number of people worldwide ( 2 ). It is the main risk factor for diabetes, hypertension, cardiovascular diseases and some types of cancer ( 2 , 3 ). As for our new enemy, SARS-CoV 2, obesity is a major risk factor for severe COVID-19 ( 3 , 4 ). Thus, we desperately seek for advances that could result in optimal prevention and treatment of obesity and comorbidities. Therefore, we must define the mechanisms that lead to the anomalous control of caloric intake and energy expenditure and how this affects distinct organs and systems in our bodies ( 5 , 6 ). Many of the answers to these questions are hidden behind the mechanisms that our body employ to respond to nutrients. In this issue of the *Archives of Endocrinology and Metabolism* , Telma Correa and cols. lead us through the most recent advances in this field ( 1 ).

The review starts with a timely definition of the term “Nutritional Genomics” and a description of some important historical aspects that led to the development of this field. We learned from these data that the Human Genome Project triggered a series of events that resulted in the identification of receptors, transcriptions factors and molecular mechanisms that are responsive to distinct components of food. Our cells are fully equipped with molecular tools employed not only to extract energy and building blocks from food, but also to regulate their function in response to signals generated by different nutrients.

Ingeniously, instead of going classic and presenting the physiological aspects of nutrigenomics, the authors go straight to the point describing how a bad choice of foods could trigger a systemic inflammatory response that is the epicenter of metabolic and cardiovascular diseases ( 7 ). In this section, they provide a very useful list of inflammatory markers that are mechanistically associated with metabolic diseases.

Once the tragedy was set, the authors offer hope by describing the benefits of the Mediterranean diet and some of its well-known components, mono and polyunsaturated fats. Here we can find out depth descriptions of the mechanisms that transduce the effects of these dietary components, ranging from receptors, intracellular messengers and genes. The section is vastly supported by original references and excellent tables.

Next, the authors discuss how genes commonly associated with obesity could be regulated by nutrients providing a broad view of the nutrient-driven regulatory mechanisms taking place in metabolic diseases.

In the end, there is a detailed description of epigenomics and the regulation of cell fate by microRNAs. This is a considerably new field that expanded our understating of how environment, including foods, modulate our genes expression and functioning on a daily basis. It is also a mechanism that explains how the parents’ good or bad choices impact on their offspring’s health.

Thus, I deeply recommend the reading of this review. For clinicians, it expands the understanding of the roles of food in human health; for residents and graduate students, it provides a huge amount of hard data that serves the purpose of education; and for the experts in the field, it is a pleasant reading that may open collaborative perspectives with this excellent group of Brazilian researchers.
